# Supplementary material for: Perceived Facilitators and Barriers for Actual Arm Use during Everyday Activities in Community Dwelling Individuals with Chronic Stroke
Source: Int J Environ Res Public Health. 2022 Sep 16;19(18):11707. doi: 10.3390/ijerph191811707 (PMC9516989; doi:10.3390/ijerph191811707)
Supplement: Supplementary file 1 [file ijerph-19-11707-s001.zip › ijerph-1897913-supplementary.pdf]

International Journal of
Environmental Research and Public Health

MDPI

Perceived Facilitators and Barriers for Actual Arm Use during Everyday Activities in Community Dwelling Individuals with Chronic Stroke

Supplementary Materials File S1

Interview Guide—Stages of Actual UE Use

Section 1

We are interested in finding out how much you actually use your affected arm during the day. Please read the definitions and answer the following questions.

First, I'm going to define some common terms used in the assessment. Keep these definitions in mind as you answer each of the SAUU questions. READ definitions below.

<u>Definitions</u>

Arm use: Not what you think you can do, but how much you actually use your affected arm to help or complete everyday tasks throughout the day. The affected arm can be used as the primary arm or as a helper. For example, during a meal, the affected arm is:

- used to hold the fork and pick up food (primary arm)
- used to stabilize the bowl or plate, while the unaffected arm holds the fork (helper).

Everyday tasks: common self-care and household activities completed throughout the day such as dressing, taking a shower, eating, using the phone, and shopping.

Regular basis: Consistent involvement of the affected arm to complete everyday tasks at least once a day. If tasks are not completed every day, the affected arm is involved in completing the task for the majority of the time (3 out of 4 times).

In the next set of questions, I'm going to ask you to rate how much you actually use your affected arm during everyday tasks, in general and for specific tasks on a scale from 0 (I presently do not use my affected arm) all the way to 4 (I have been using my affected arm regularly for 3 months or more). First give me the rating for each question, then I will ask you what you are thinking about as you answer each question.

**Instructions**: Please circle the number that best describes how much you use your arm to assist or complete **everyday tasks in general** throughout the day.		**Cognitive Interview Probe: Tell me what you are thinking about as you answer the question**
**General Arm Use**	**Stage**	
I presently use my affected arm regularly and have for more than 3 months.	4	Please repeat this question in your own words
I presently use my affected arm regularly but have for less than 3 months.	3	Why do you think you use your arm…
I presently use my affected arm at times but not regularly.	2	How sure you are that you use your arm…
I presently do not use my affected arm but think about starting within 3 months.	1	How do you remember that you use your arm…
I presently do not use my affected arm and do not plan to start in the next 3 months.	0	How did you arrive at that answer?<br>What it easy or hard to answer

**Instructions**: Mark the box that best describes **actual use** of your affected arm for each task.						
Everyday task	4	3	2	1	0	**Cog interview Probe: Tell me what you are thinking about as you answer each question**
Dressing (upper and lower body)						Please repeat this question in your own words<br>Why do you think you use your arm…?<br>How sure are you that you use your arm xxx when dressing?<br>How do you remember that you use your arm xxx?<br>How did you arrive at that answer?<br>Was that easy or hard to answer?
Grooming (e.g., brushing teeth, washing face and hands)						Please repeat this question in your own words<br>Why do you think you use your arm xx during xxx?<br>How sure are you that you use your arm xxx?<br>How do you remember that you use your arm xxx?<br>How did you arrive at that answer?<br>Was that easy or hard to answer?
Toileting (toilet paper, perineal care)						Please repeat this question in your own words<br>Why do you think you use your arm xx during xxx?<br>How sure are you that you use your arm xxx?<br>How do you remember that you use your arm xxx?<br>How did you arrive at that answer?<br>Was that easy or hard to answer?

Laundry (e.g., using washer/dryer, folding clothes)	Please repeat this question in your own words<br>Why do you think you use your arm xx during xxx?<br>How sure are you that you use your arm xxx?<br>How do you remember that you use your arm xxx?<br>How did you arrive at that answer?<br>Was that easy or hard to answer?
Bathing/showering	Please repeat this question in your own words<br>Why do you think you use your arm xx during xxx?<br>How sure are you that you use your arm xxx?<br>How do you remember that you use your arm xxx?<br>How did you arrive at that answer?<br>Was that easy or hard to answer?
Eating (e.g., using utensils, opening containers)	Please repeat this question in your own words<br>Why do you think you use your arm xx during xxx?<br>How sure are you that you use your arm xxx?<br>How do you remember that you use your arm xxx?<br>How did you arrive at that answer?<br>Was that easy or hard to answer?
Meal preparation (e.g., getting drink from refrigerator, making sandwich)	Please repeat this question in your own words<br>Why do you think you use your arm xx during xxx?<br>How sure are you that you use your arm xxx?<br>How do you remember that you use your arm xxx?<br>How did you arrive at that answer?<br>Was that easy or hard to answer?
Medication management (e.g., opening bottle or pill box, taking pills)	Please repeat this question in your own words<br>Why do you think you use your arm xx during xxx?<br>How sure are you that you use your arm xxx?<br>How do you remember that you use your arm xxx?<br>How did you arrive at that answer?<br>Was that easy or hard to answer?
Shopping (e.g., pushing cart, grabbing item from shelf, holding bags)	Please repeat this question in your own words<br>Why do you think you use your arm xx during xxx?<br>How sure are you that you use your arm xxx?<br>How do you remember that you use your arm xxx?<br>How did you arrive at that answer?<br>Was that easy or hard to answer?
Mobile technology-smartphone, iPad (e.g., texting, making calls, emailing)	Please repeat this question in your own words<br>Why do you think you use your arm xx during xxx?<br>How sure are you that you use your arm xxx?<br>How do you remember that you use your arm xxx?<br>How did you arrive at that answer?<br>Was that easy or hard to answer?

Section 2

In this section, we would like you to think about how confident you feel about using your affected arm in different situations and rate each question on rating scale between 1 (not confident) to 4 (very confident).

Instruction: Mark the box that best describes how confident you feel in the following situations.

"I am confident I can integrate my affected arm in bilateral tasks (tasks that require two hands) when I am..."

	**1: Not confident**	**2: Somewhat confident**	**3: Fairly confident**	**4: Very confident**	**Cog interview Probe: Tell me what you are thinking about as you answer each question**
Tired					Please repeat this question in your own words<br>Why do you think you are xx when you are tired?<br>How sure are you that you feel this way?<br>How do you remember that you are xxx?<br>Was that easy or hard to answer?
In a bad mood/depressed					Please repeat this question in your own words<br>Why do you think you are xxx about using your arm when you are in a bad mood or feel depressed?<br>How sure are you that you feel this way?<br>How do you remember that you xxx?<br>How did you arrive at that answer?<br>Was that easy or hard to answer?
In a rush					Please repeat this question in your own words<br>Why do you think you are xxx about using your arm when you are in a rush?<br>How sure are you that you feel this way?<br>How do you remember that you are xxx when you're in a rush?<br>How did you arrive at that answer?<br>Was that easy or hard to answer?
In public places unfamiliar to me					Please repeat this question in your own words<br>Why do you think you are xxx about using your arm when you are in public spaces or unfamiliar places?<br>How sure are you that you feel this way?<br>How do you remember that you are xxx when you're in unfamiliar places?<br>How did you arrive at that answer?<br>Was that easy or hard to answer?
In discomfort or my arm feels stiff					Please repeat this question in your own words<br>Why do you think you xxx about using your arm when you are in discomfort or feel still?<br>How sure are you that you feel this way?<br>How do you remember that you xxx when you're feeling discomfort or stiffness?<br>How did you arrive at that answer?<br>Was that easy or hard to answer?
At work or volunteering					Please repeat this question in your own words<br>Why do you think you are xxx about using your arm at work or when volunteering?<br>How sure are you that you feel this way?

	How do you remember that you are xxx at work or when volunteering?<br>How did you arrive at that answer?<br>Was that easy or hard to answer?<br>Please repeat this question in your own words
Trying to do a new activity	Why do you think you are xxx when using your arm to try a new activity?<br>How sure are you that you feel this way?<br>How do you remember that you are xxx when trying a new activity?<br>How did you arrive at that answer?<br>Was that easy or hard to answer?<br>Please repeat this question in your own words
By myself, and don't have the help of family or friends	Why do you think you are xxx when using your arm when you're by yourself?<br>How sure are you that you that you feel this way?<br>How do you remember that you are xxx when you're by yourself?<br>How did you arrive at that answer?<br>Was that easy or hard to answer?
